# Adaptive second-order backstepping control for a class of 2DoF underactuated systems with input saturation and uncertain disturbances

**DOI:** 10.1038/s41598-024-66552-6

**Published:** 2024-07-09

**Authors:** Weiping Guo, Diantong Liu

**Affiliations:** School of Computer and Control Engineering, Yantai University, Yantai, 264005 Shandong China

**Keywords:** Mathematics and computing, Electrical and electronic engineering, Mechanical engineering

## Abstract

An adaptive second-order backstepping control algorithm is proposed for a kind of two degrees of freedom (2DoF) underactuated systems. The system dynamics is transformed into a nonlinear feedback cascade system with an improved global change of coordinates. Fully taking the cascade structure into consideration and in order to simplify the design process, each step in the backstepping process is designed for a second-order subsystem. Two neural networks are applied to approximate system unknown functions and two adaptive laws are designed to estimate the upper bound of the sum of approximation error and external disturbances. To overcome the explosion problem of complexity, a second-order filter is applied to produce the virtual control and its second-order derivative that is needed in the next backstepping step. Two auxiliary dynamic systems are proposed and integrated into the backstepping process to eliminate the effects of filtering error and input saturation. The system stability is analyzed by the Lyapunov stability theory and verified by numerical simulations with two 2DoF benchmark underactuated systems: the translational oscillator with a rotational actuator (TORA) and the inertial wheel pendulum (IWP).

## Introduction

Underactuated systems are a class of nonlinear systems that have fewer control inputs than the degrees of freedom (DoF)^1^. For an underactuated system, the control inputs can only directly control part of its DoFs, and the other DoFs are handled through the system’s internal dynamics, and it may exhibit weak nonlinear interaction or a high nonlinearity between the controlled and uncontrolled DoFs^2^. Some mature control technologies for nonlinear systems can’t be directly used, such as feedback linearization and backstepping. At the same time, the underactuated systems are widely used in engineering practice so as to reduce body weight, failure rate, energy consumption, and manufacturing cost^3^. Besides the wide practical applications, it is essential and exciting for researchers to test their proposed nonlinear control algorithm with the underactuated systems as prototype systems.

The wide application and important theoretical significance of underactuated systems make many scholars focus on the control of underactuated systems, and some control technologies have been proposed in the literature. Some scholars focus on intelligent control algorithms, and some scholars focus on nonlinear control algorithms. For the intelligent control algorithms, the fuzzy or fuzzy-based control algorithms are proposed^4,5^, and neural network (NN) or NN-based controllers are proposed^6–8^. For the nonlinear control algorithms, the partial feedback linearization^1^, the adaptive control methods^7,9^, the energy- or passivity-based control methods^10,11^, the robust control methods^12,13^, and the sliding mode-based methods^14,15^ are proposed. Despite the control classifications made for the above literature, almost all are a combination of multiple control algorithms.

There is much research work on underactuated systems, which mainly focuses on a specific underactuated object^16^, such as TORA, Acrobat, IWP, inverted pendulum, etc. On the other hand, there are several general methods to analyze or control a class of underactuated systems: A kind of classification^2^ has been proposed for 2DoF underactuated systems based on some system structural properties, such as integrable normalized generalized momentums, kinetic symmetry, actuation mode, and interacting inputs. The proposed cascade normal forms in a feedback or feedforward form, make the underactuated systems possible to design with existing backstepping and feedforwarding procedures. Some attempts are made for some underactuated systems with the backstepping—a systematic recursive design method. The system model^17^ is transformed into a cascaded model that is composed of a core nonlinear subsystem and a second-order linear subsystem. A nonlinear controller^2^ is proposed to control the core subsystem, and a controller is designed with the traditional backstepping method to control the second-order linear subsystem. The strict-feedback form is obtained with a novel coordinate transformation and the backstepping control is proposed^18^. A defined control variable is chosen to start the backstepping steps^19^, and the design steps depend too much on the control variable.

As we all know, the drawback of the classic backstepping design method is the explosion of complexity (EoC), which leads to computational complexity and burden in the recursive design process. To handle the EoC problems, the dynamic surface control (DSC)^20^ is proposed, in which a first-order filter is applied in each step to allow a design where the model is not differentiated. There have been reports on DSC schemes^21,22^, and some DSC solutions^17,23^ are designed for the underactuated systems. However, the uncompensated filtering errors may affect the control performance. Recently, the command filters^24,25^ are proposed to eliminate the effects of filter errors with auxiliary dynamics in the backstepping process.

In practical control engineering, input saturation is common and inevitable, which can reduce system control performance, and even lead to closed-loop instability or control failure. Many significant results on input saturation have been reported, and there are two main categories. The first^26^ is to replace the nonsmooth nonlinear saturation function with a hyperbolic tangent function, and the inconsistency between the two functions isn’t handled in the design process, which may reduce the system control performance. The second^27^ is to use the auxiliary dynamic system to compensate for the inconsistency between with and without input saturation, and the auxiliary dynamics is directly used to obtain the controller. In addition, external disturbance and parameter uncertainty can also affect the control performance and even lead to instability. To solve this problem, some attempts are performed with disturbance observer^28,29^ and NN disturbance estimator^30^.

For a class of 2DoF underactuated systems, some control attempts have been performed, such as continuous higher-order sliding-mode-control^12^, dynamic surface control^17^, RISE control^31^, in which the input saturation, external interference, inertial parameter uncertainty are not considered. For the TORA, which belongs to underactuated systems, a command filter controller is proposed in our research^32^ and the bounded torques are replaced by the hyperbolic tangent function input^33^.

Inspired by the above research work, an adaptive second-order backstepping control algorithm is proposed for a class of 2DoF underactuated systems with inertial parameter uncertainties, external disturbances, and input saturation. To begin the backstepping process, the class of 2DoF underactuated systems is transformed into a nonlinear cascade model in the feedback form with an improved global coordinate change. In the backstepping process, the two subsystems in the cascade model are considered as a whole. That is to say, either the virtual control signal or the control signal is designed for a second-order subsystem. Thus, only two steps are needed in the backstepping process for a 2DoF underactuated system. From the view of control engineering, there usually exist input saturation, internal uncertainties, external disturbances, and unknown system functions in the system dynamics. It is still a challenging problem to control the underactuated systems with input saturation and uncertain disturbances. Two neural networks are applied to approximate the system functions and two adaptive laws are designed to estimate the upper bound of the sum of NN approximation error and uncertain disturbances. A second-order filter is used to produce virtual control and its second-order derivative, accompanied by a dynamic compensation system for filtering errors. Moreover, another auxiliary dynamics are constructed to eliminate the effect of input saturation. The main contributions and significance of this paper are summarized as follows:

1. To the best of our knowledge, this is the first attempt to systematically design an adaptive backstepping controller and theoretically prove the stability for a class of 2DoF underactuated systems with input saturation and uncertain disturbances.
2. The second-order backstepping idea, i.e. every backstepping step is designed for a second-order subsystem, is proposed to reduce the number of design steps and simplify the design process. To overcome the EoC problems, a second-order filter is integrated to replace the second-order derivative that is needed in the next step.
3. In the backstepping process, the NNs and adaptive laws are designed to approximate the unknown system functions and the upper bound of system uncertainties. Two auxiliary dynamic systems are proposed to eliminate the effects of filtering error and input saturation.

The structure of this paper is as follows. "System description" is the system description for a class of 2DoF underactuated systems and the dynamics are dealt with an improved global coordinate change to obtain its feedback cascade model."Adaptive second-order backstepping control design", the system error dynamics are described, the adaptive second-order backstepping control is designed in detail, and the system stability is analyzed. Some numerical simulations are performed with TORA and IWP to validate the system stability and control performance of the proposed control algorithm in "Numerical simulations" and "Conclusions" is the conclusions.

## System description

A class of 2DoF underactuated system described in

**Definition 3.9.1**^2^ is considered in this section, which has two DoFs with configuration vector $q=(q_1, q_2)^{T}$, and its positive definite symmetric inertia matrix only depends on $q_2$, i.e. $M=M(q_2)$. The Lagrangian of the class of underactuated systems can be expressed as follows.

1 $$\begin{aligned} L(q,\dot{q})=\frac{1}{2}\dot{q}^T\left[ \begin{array}{l} m_{11}(q_2)\quad m_{12}(q_2)\\ m_{11}(q_2)\quad m_{12}(q_2) \end{array} \right] \dot{q}-V(q) \end{aligned}$$

where *V*(*q*) is the system’s potential energy.

The Euler-Lagrange equations of motion can be built as

2 $$\begin{aligned} \left\{ \begin{array}{l} m_{11}(q_2)\ddot{q}_1+m_{12}(q_2)\ddot{q}_2+m'_{11}(q_2)\dot{q_1}\dot{q_2} +m'_{12}(q_2)\dot{q}^2_2-g_1(q_1,q_2)=d_1 \\ m_{21}(q_2)\ddot{q}_1+m_{22}(q_2)\ddot{q}_2-\frac{1}{2}m'_{11}(q_2)\dot{q}^2_1+\frac{1}{2}m'_{22}(q_2)\dot{q}^2_2-g_2(q_1,q_2)=\tau +d_2 \end{array} \right. \end{aligned}$$

where, $\tau$ is the control input, $g_{i}(q_{1},q_{2})=-\partial {V}(q)/\partial {q_{i}}, i=1,2$ and $\prime$ denotes $d/dq_{2}$. $d_{1}$ and $d_{2}$ respectively are the unknown external disturbances (including frictions and other external disturbances) acting on each DoF. The properties of the system described by Eq. (2) can be found in detail^2,6,12^, and some properties are beneficial for the design and analysis of the closed-loop control system^6^.

The following improved global coordinate changes

3 $$\begin{aligned} \left\{ \begin{array}{l} x_1=q_1+\displaystyle {\int _{0}^{q_2}}\dfrac{m_{12}(s)}{m_{11}(s)}ds\\ x_2=\dot{q}_1+\dfrac{m_{12}(q_2)}{m_{11}(q_2)}\dot{q}_2\\ x_3=q_2\\ x_4=\dot{q}_2 \end{array} \right. \end{aligned}$$

can transform the system dynamics Eq. (2) into the following nonlinear model

4 $$\begin{aligned} \left\{ \begin{array}{l} \dot{x}_{1}=x_{2} \\ \dot{x}_2=f_1(x_1, x_2, x_3)+x_3+\overline{d}_1 \\ \dot{x}_3=x_4 \\ \dot{x}_4=f_2(x_1, x_2, x_3, x_4, \tau )+\tau +\overline{d}_2 \end{array} \right. \end{aligned}$$

where,

$$\begin{aligned} & f_{1} (x_{1} ,x_{2} ,x_{3} ) = \frac{{g_{1} (q_{1} ,q_{2} )}}{{m_{{11}} (q_{2} )}} - x_{3} , \\ & \bar{d}_{1} = \left( {g_{{d1}} (q_{2} ) + \Delta g_{{d1}} (q_{2} )} \right)d_{1} + f_{m} (x_{1} ,x_{2} ,x_{3} ,x_{4} ) + \Delta f_{m} (x_{1} ,x_{2} ,x_{3} ,x_{4} ) + \Delta f_{1} (x_{1} ,x_{3} ), \\ & g_{{d1}} (q_{2} ) = \left( {m_{{11}} (q_{2} )} \right)^{{ - 1}} ,\quad f_{m} (x_{1} ,x_{2} ,x_{3} ,x_{4} ) = - \frac{{m_{{11}} (q_{2} )m^{\prime}_{{11}} (q_{2} )\mathop {q_{1} }\limits^{.} \mathop {q_{2} }\limits^{.} + m^{\prime}_{{11}} (q_{2} )m_{{12}} (q_{2} )\dot{q}_{2}^{2} }}{{m_{{11}}^{2} (q_{2} )}}, \\ & f_{2} (x_{1} ,x_{2} ,x_{3} ,x_{4} ,\tau ) = g_{4} (q_{2} )\left( {m_{{21}} (q_{2} )m^{\prime}_{{11}} (q_{2} )\dot{q}_{1} \dot{q}_{2} } \right. + m_{{21}} (q_{2} )m^{\prime}_{{12}} (q_{2} )\dot{q}_{2}^{2} - m_{{21}} (q_{2} )g_{1} (q_{1} .q_{2} ) + m_{{11}} (q_{2} )g_{2} (q_{1} ,q_{2} ) \\ & \quad + \frac{1}{2}m_{{11}} (q_{2} )m^{\prime}_{{11}} (q_{2} )\dot{q}_{1}^{2} - \frac{1}{2}m_{{11}} (q_{2} )m^{\prime}_{{22}} (q_{2} )\dot{q}_{2}^{2} ) + (m_{{11}} (q_{2} )g_{4} (q_{2} ) - 1)\tau , \\ & g_{4} (q_{2} ) = \left( {m_{{11}} (q_{2} )m_{{22}} (q_{2} ) - m_{{12}} (q_{2} )m_{{21}} (q_{2} )} \right)^{{ - 1}} , \\ & \bar{d}_{2} = (g_{{d2(q_{2} )}} + \Delta g_{{d2}} (q_{2} ))d_{2} + (g_{{d3}} (q_{2} ) + \Delta g_{{d3}} (q_{2} ))d_{1} + \Delta f_{2} (x_{1} ,x_{2} ,x_{3} ,x_{4} ,\tau ), \\ & g_{{d2}} (q_{2} ) = g_{4} (q_{2} )m_{{11}} (q_{2} ),\quad g_{{d3}} (q_{2} ) = - g_{4} (q_{2} )m_{{21}} (q_{2} ). \\ \end{aligned}$$

with

5 $$\begin{aligned} \left\{ \begin{array}{l} q_1=x_1-\displaystyle {\int _{0}^{x_3}}\dfrac{m_{12}(s)}{m_{11}(s)}ds\\ \dot{q}_1=x_2-\dfrac{m_{12}(x_3)}{m_{11}(x_3)}x_4\\ q_2=x_3\\ \dot{q}_2=x_4 \end{array} \right. \end{aligned}$$

$\Delta {f}_1(x_1,x_2,x_3)$, $\Delta {f}_2(x_1,x_2,x_3,x_4,\tau )$, $\Delta {g}_{d1}(x_3)$, $\Delta {g}_{d2}(x_3)$ and $\Delta {g}_{d3}(x_3)$ are the uncertain functions due to the internal parameter uncertainties , $\overline{d}_1$ and $\overline{d}_2$ are the equivalent uncertain disturbances on unactuated and actuated DoFs after global coordinate changes.

For a practical system, the actuator output usually is limited. The saturation function can be described as follows.

6 $$\begin{aligned} \tau =\tau (u)=\left\{ \begin{array}{l} u_{min}, \quad \quad u<u_{min} \\ u, \ \quad \quad \quad u_{min}\le u\le u_{max} \\ u_{max}, \quad \quad u>u_{max} \\ \end{array} \right. \end{aligned}$$

where *u* is the control input that is calculated by the to-be-proposed control algorithm. $u_{max} > 0$ and $u_{min} < 0$ are the known amplitudes of input saturation.

### Assumption 1

It is assumed that the amplitudes of input saturation, parameters uncertainties, and external disturbances are bounded, which implies $|\overline{d}_1|\le \overline{d}^M_1$ and $|\overline{d}_2|\le \overline{d}^M_2$ .

### Assumption 2

It is assumed that the position and velocity can be obtained with an encoder and tachometer for each DoF^6^.

In this paper, a class of 2DoF underactuated systems with the dynamics model Eq. (2) is considered and it can be transformed through a global change of coordinates Eq. (3) into the cascaded form Eq. (4), where, $x=[x_1,x_2,x_3,x_4]$ is the state vector, $\tau$ is the control input, $f_1, f_2$ are unknown functions, and $\overline{d}_1, \overline{d}_2$ are equivalent unknown disturbances that are composed of parameter uncertainties and external disturbance acting on each DoF. The control objective is to design a nonlinear controller to stabilize the 2DoF underactuated system to its origin with input saturation and uncertain disturbances.

### Remark 1

It can be seen from Eq. (4) that the theoretical model of the class of 2DoF underactuated systems can be written as a nonlinear cascade model in the feedback form when $x_3$ in the second equation is looked at as a disturbance in $\overline{d}_1$. For some 2DoF underactuated systems, such as TORA and IWP, the $m_{11}(q_2)$ in inertia matrix $M(q_2)$ is independent of $q_2$ and $m'_{11}(q_2)=0$ makes Eq. (4) a true nonlinear cascade system in the feedback form.

### Remark 2

The dynamics of the first subsystem $(x_1, x_2)$ are driven by $x_3$, which can be looked at as the output of the second subsystem $(x_3, x_4)$ driven by $\tau$. That is to say, using $x_3$ as the virtual control signal of the first subsystem and $\tau$ as control signal of the second subsystem, it is possible to globally stabilize the system dynamics to its origin through the backstepping technique, in which each step is designed for a second-order subsystem, i.e. second order backstepping. From the view of application, the two variables of each subsystem belong to the same DoF, the second-order backstepping design can improve the system performance through adjusting the parameters in controller.

### Remark 3

In order to make $x_2$ equal to the differential of $x_1$, the global coordinate changes Eq. (3) are improved from the global coordinate changes in Proposition 3.9.1^2^ , which can make the auxiliary systems in the next section have the same structure as the system dynamics Eq. (4) and make it easy to obtain the error dynamics of the system. Moreover, this kind of structure makes the second-order backstepping design easier to implement.

### Remark 4

For some actual underactuated systems, the interaction between different DoFs is weak or sinusoid-type nonlinear and there is no affine appearance, so the system dynamics are looked as a pure feedback form in the design process. The two nonlinear functions $f_1(x_1,x_2, x_3)$ and $f_2(x_1,x_2, x_3, x_4, \tau )$ in Eq. (4) may be unknown nonlinear functions since that the modeling terms including $m_{11}(q_2)$, $m_{12}(q_2)$, $m_{21}(q_2)$, $m_{22}(q_2)$, and *V*(*q*) in Eq. (1) maybe can’t be accurately obtained. Moreover, the terms $\overline{d}_i$, $i=1,2$ in Eq. (4) are transformed from internal parameters uncertainties and external disturbances, and they also are functions of the states so their variation bounds are also unknown.

## Adaptive second-order backstepping control design

Since the global change of coordinates Eq. (3) has transformed the theoretical model of the class of underactuated systems Eq. (2) into a nonlinear cascade model in the feedback form as Eq. (4), the backstepping steps can be performed to obtain a nonlinear controller.

In this section, Two RBF neural networks are applied to approximate the functions $f_1(x_1,x_2, x_3)$ and $f_2(x_1,x_2, x_3, x_4, \tau )$ in Eq. (4) and two adaptive laws are designed to estimate the upper bound of the sum of approximation error and external disturbances, which will be used in the backstepping process. Moreover, a second-order filter will be applied to produce the virtual control signal and its second-order derivative to overcome the EoC. Two auxiliary dynamic systems will be integrated into the second-order backstepping process to eliminate the effects of the second-order filtering error and input saturation. A nonlinear controller will be obtained with second-order backstepping.

### The system error dynamics

In the design process, the following second-order filter^24,34^ is used.

7 $$\begin{aligned} \left\{ \begin{array}{l} \dot{x}_{3c}=x_{4c} \\ \dot{x}_{4c}=-2\zeta \omega _nx_{4c}-\omega ^2_n(x_{3c}-x_{3d}) \end{array} \right. \end{aligned}$$

where, $\zeta \in (0, 1]$ and $\omega _n$ are the positive design parameters, $x_{3d}$ is the input of the second-order filter and the virtual control signal designed later, $x_{3c}$ and $\ddot{x}_{3c}$ (i.e.$\dot{x}_{4c}$) are the second-order filter outputs and will be used in the following control design process. The initial conditions are $x_{3c}(0)=x_{3d}(0)$ and $x_{4c}(0)=0$.

Considering that the filtering errors arise with the second-order filter and will affect the system control performance, the compensating signals $\xi _1$ and $\xi _2$ are adopted to eliminate the impact of errors $x_{3c}-x_{3d}$. The auxiliary dynamic system are designed as

8 $$\begin{aligned} \left\{ \begin{array}{l} \dot{\xi }_1=\xi _2 \\ \dot{\xi }_2=-k_1\xi _1-k_2\xi _2+(x_{3c}-x_{3d}) \end{array} \right. \end{aligned}$$

where, $k_1$ and $k_2$ are the positive design parameters, the initial conditions are $\xi _1(0)=0$ and $\xi _2(0)=0$.

In order to eliminate the effect of input saturation on system performance, the following auxiliary dynamic system is designed to generate the compensation signals $\varsigma (t)=[\varsigma _1,\varsigma _2,\varsigma _3,\varsigma _4]^T$

9 $$\begin{aligned} \left\{ \begin{array}{l} \dot{\varsigma }_1=\varsigma _2 \\ \dot{\varsigma }_2=-k_1\varsigma _1-k_2\varsigma _2+\varsigma _3\\ \dot{\varsigma }_3=\varsigma _4 \\ \dot{\varsigma }_4=-k_3\varsigma _3-k_4\varsigma _4+\tau -u \end{array} \right. \end{aligned}$$

where $k_3$ and $k_4$ are the positive design parameters. The initial consitions are $\varsigma _1(0)=0$, $\varsigma _2(0)=0$, $\varsigma _3(0)=0$, $\varsigma _4(0)=0$.

The control objective is to stabilize the 2DoF underactuated systems so as to make system states converge to their equilibrium position. After the filter compensation Eq.(8) and input saturation compensation Eq. (9), the system errors $e(t)=[e_1, e_2, e_3, e_4]^T$ are defined as

10 $$\begin{aligned} \left\{ \begin{array}{l} e_1=x_1-\xi _1-\varsigma _1 \\ e_2=x_2-\xi _2-\varsigma _2 \\ e_3=x_3-x_{3c}-\varsigma _3 \\ e_4=x_4-x_{4c}-\varsigma _4 \end{array} \right. \end{aligned}$$

Thus, the system error dynamics can be obtained from Eqs. (4), (7)–(10)

11 $$\begin{aligned} \left\{ \begin{array}{l} \dot{e}_1=e_2 \\ \begin{aligned} \dot{e}_2=&{}f_1(x_1,x_2,x_3)+x_3+\overline{d}_1+k_1\xi _1+k_2\xi _2-(x_{3c}-x_{3d})+k_1\varsigma _1+k_2\varsigma _2-\varsigma _3 \end{aligned}\\ \dot{e}_3=e_4 \\ \begin{aligned} \dot{e}_4=&{}f_2(x_1, x_2, x_3,x_4,\tau )+\overline{d}_2-\dot{x}_{4c}+k_3\varsigma _3+k_4\varsigma _4+u \end{aligned}\\ \end{array} \right. \end{aligned}$$

### The second-order backstepping design

The design process is as follows.

*Step 1.*

Firstly, the first second-order subsystem described by $(e_1, e_2)$ in the system error dynamics Eq. (11) is considered. The error states $e_1$ and $e_2$ are rewritten as

12 $$\begin{aligned} \left\{ \begin{array}{l} \dot{e}_1=e_2 \\ \begin{aligned} \dot{e}_2=&{}f_1(x_1,x_2,x_3)+x_3+\overline{d}_1+k_1\xi _1+k_2\xi _2-(x_{3c}-x_{3d})+k_1\varsigma _1+k_2\varsigma _2-\varsigma _3 \end{aligned} \end{array} \right. \end{aligned}$$

Due to the existence of unknown function $f_1(x_1,x_2, x_3)$ and equivalent unknown disturbance $\overline{d}_1$, the virtual control signal $x_{3d}$ cannot be obtained directly. In this research, $f_1(x_1,x_2, x_3)$ is approximated by a RBF NN $\theta ^{*T}_1\varphi _1(x_1,x_2,x_3)$ consequently, given a compact set $\Omega _1\in {R^3}$ , let $\theta ^{*T}_1$ and $\varepsilon _1^*$ be such that for any $(x_1,x_2, x_3)\in \Omega _1$

13 $$\begin{aligned} f_1(x_1,x_2, x_3)=\theta ^{*T}_1\varphi _1(x_1,x_2, x_3)+ \varepsilon ^*_1 \end{aligned}$$

where, $\theta ^{*}_1$ is the ideal weights vector and its upper bound is $|\theta ^{*}_1|\le \theta ^{M}_1$. $\varepsilon ^*_1$ shows the approximation error and its upper bound is $|\varepsilon ^*_1|\le \varepsilon ^M_1$.

It can be obtained from Eqs. (12) and (13)

14 $$\begin{aligned} \begin{aligned} \dot{e}_2=&\theta ^{*T}_1\varphi _1(x_1,x_2, x_3)+\varepsilon ^*_1+\overline{d}_1+x_3+k_1\xi _1+k_2\xi _2-(x_{3c}-x_{3d})+k_1\varsigma _1+k_2\varsigma _2-\varsigma _3 \end{aligned} \end{aligned}$$

Choose the virtual control $x_{3d}$ as follows

15 $$\begin{aligned} \begin{aligned} x_{3d}=&-k_1x_1-k_2x_2-\theta ^{T}_1\varphi _1(x_1,x_2, x_3)-\chi _1tanh\left( \frac{p_{12}e_1+p_{14}e_2}{\mu _{\chi _1}}\right) \end{aligned} \end{aligned}$$

where $\theta _1$ is the estimation of $\theta ^{*}_1$ and the estimation error is $\widetilde{\theta }_1=\theta ^{*}_1-\theta _1$. $\chi _1$ is the estimation of $\chi ^M_1=\varepsilon ^{M}_1+\overline{d}^M_1$ and the estimation error is $\widetilde{\chi }_1=\chi ^M_1-{\chi }_1$, $\mu _{\chi _1}, p_{12}$ and $p_{14}$ are positive design constants.

The system error $e_2$ dynamics can be obtained from Eqs. (14) and (15).

16 $$\begin{aligned} \begin{aligned} \dot{e}_2&=\theta ^{*T}_1\varphi _1(x_1,x_2, x_3)+\varepsilon ^*_1+\overline{d}_1+x_3-x_{3d}-k_1x_1-k_2x_2-\theta ^{T}_1\varphi _1(x_1, x_3)-\chi _1tanh\left( \frac{p_{12}e_1+p_{14}e_2}{\mu _{\chi _1}}\right) \\&\quad +k_1\xi _1+k_2\xi _2-(x_{3c}-x_{3d})+k_1\varsigma _1+k_2\varsigma _2-\varsigma _3\\&=-k_1e_1-k_2e_2+e_3+ \widetilde{\theta }^T_1\varphi _1(x_1,x_2, x_3)+\varepsilon ^*_1+\overline{d}_1-\chi _1tanh\left( \frac{p_{12}e_1+p_{14}e_2}{\mu _{\chi _1}}\right) \end{aligned} \end{aligned}$$

The first sencond-order subsystem errors $(e_1, e_2)$ dynamics after compensations can be rewritten together as

17 $$\begin{aligned} \left\{ \begin{array}{l} \dot{e}_1=e_2 \\ \begin{aligned} \dot{e}_2=&{}-k_1e_1-k_2e_2+e_3+ \widetilde{\theta }^T_1\varphi _1(x_1,x_2, x_3)+\varepsilon ^*_1+\overline{d}_1-\chi _1tanh\left( \frac{p_{12}e_1+p_{14}e_2}{\mu _{\chi _1}}\right) \end{aligned} \end{array} \right. \end{aligned}$$

where, $k_1$ and $k_2$ are chosen such that

18 $$\begin{aligned} \ddot{\vartheta }+k_2\dot{\vartheta }+k_1\vartheta =0 \end{aligned}$$

is asymptotically stable at $(\vartheta ,\dot{\vartheta })=(0, 0)$.

Equation 17) canbe written in matrix form,

19 $$\begin{aligned} \begin{aligned} \dot{e}_{12}&=A_1e_{12}+B_1\left( e_3+ \widetilde{\theta }^T_1\varphi _1(x_1,x_2, x_3)+\varepsilon ^*_1+\overline{d}_1\right) -B_1\chi _1tanh\left( \frac{p_{12}e_1+p_{14}e_2}{\mu _{\chi _1}}\right) \end{aligned} \end{aligned}$$

where, $$e_{12}=\left[ \begin{array}{l}e_1 \\ e_2\end{array}\right]$$, $$A_1=\begin{bmatrix} 0 &{} 1\\ -k_1 &{} -k_2\end{bmatrix}$$, $$B_1=\left[ \begin{array}{l}0 \\ 1\end{array}\right]$$.

According to the idea of control theory for linear systems, two positive parameters $k_1$ and $k_2$ can be chosen to make sure that the following two positive definite symmetry matrices exist.

$$\begin{aligned} P_1=\begin{bmatrix}p_{11}&{}p_{12} \\ p_{12} &{}p_{14}\end{bmatrix},\quad Q_1=\begin{bmatrix}q_{11}&{}0 \\ 0 &{}q_{14}\end{bmatrix} \end{aligned}$$

so that

20 $$\begin{aligned} A^T_1P_1+P_1A_1=-Q_1 \end{aligned}$$

The Lyapunov function is chosen as follows:

21 $$\begin{aligned} V_1=\dfrac{1}{2}e^T_{12}P_1e_{12}+ \dfrac{1}{2}\widetilde{\theta }^T_1\Gamma ^{-1}_{\theta _1}\widetilde{\theta }_1+ \dfrac{1}{2}\widetilde{\chi }^T_1\Gamma ^{-1}_{\chi _1}\widetilde{\chi }_1 \end{aligned}$$

where $\Gamma _{\theta _1}$ and $\Gamma _{\chi _1}$ are positive definite matrices. The time derivative $\dot{V}_1$ is given by

$$\begin{aligned} \dot{V}_1&=-\dfrac{1}{2}e^T_{12}Q_1e_{12}+e^T_{12}P_1B_1\bigg (e_3+\widetilde{\theta }^T_1\varphi _1(x_1,x_2, x_3)+\varepsilon ^*_1+\overline{d}_1\bigg )-e^T_{12}P_1B_1\chi _1tanh\left( \frac{p_{12}e_1+p_{14}e_2}{\mu _{\chi _1}}\right) \\&\quad +\widetilde{\theta }^T_1\Gamma ^{-1}_{\theta _1}\dot{\widetilde{\theta }}_1+\widetilde{\chi }^T_1\Gamma ^{-1}_{\chi _1}\dot{\widetilde{\chi }}_1 \\&=-\dfrac{1}{2}e^T_{12}Q_1e_{12}+e^T_{12}P_1B_1e_3+e^T_{12}P_1B_1\left( \varepsilon ^*_1+\overline{d}_1\right) +\widetilde{\theta }^T_1\Gamma ^{-1}_{\theta _1}\left( \Gamma _{\theta _1}e^T_{12}P_1B_1\varphi _1(x_1,x_2, x_3)-\dot{\theta }_1\right) \\&\quad -e^T_{12}P_1B_1\chi _1tanh\left( \frac{p_{12}e_1+p_{14}e_2}{\mu _{\chi _1}}\right) +\widetilde{\chi }^T_1\Gamma ^{-1}_{\chi _1}\dot{\widetilde{\chi }}_1\\&\le -\dfrac{1}{2}q_{11}e^2_1-\dfrac{1}{2}q_{14}e^2_2+(p_{12}e_1+p_{14}e_2)e_3+|p_{12}e_1+p_{14}e_2|\chi ^{M}_1\\&\quad -(p_{12}e_1+p_{14}e_2)\chi ^M_1tanh\left( \frac{p_{12}e_1+p_{14}e_2}{\mu _{\chi _1}}\right) \\&\quad +\widetilde{\theta }^T_1\Gamma ^{-1}_{\theta _1}\left( \Gamma _{\theta _1}e^T_{12}P_1B_1\varphi _1(x_1, x_3)-\dot{\theta }_1\right) +\widetilde{\chi }^T_1\Gamma ^{-1}_{\chi _1}\bigg ((p_{12}e_1+p_{14}e_2)\Gamma _{\chi _1}tanh\left( \frac{p_{12}e_1+p_{14}e_2}{\mu _{\chi _1}}\right) -\dot{{\chi }}_1\bigg ) \end{aligned}$$

The parameter updating laws $\dot{\theta }_1$ and $\dot{{\chi }}_1$ are designed as

22 $$\begin{aligned} \dot{\theta }_1= & {} (p_{12}e_1+p_{14}e_2)\Gamma _{\theta _1}\varphi _1(x_1, x_3)-\eta _{\theta _1}\Gamma _{\theta _1}\theta _1 \end{aligned}$$

23 $$\begin{aligned} \dot{{\chi }}_1= & {} (p_{12}e_1+p_{14}e_2)\Gamma _{\chi _1}tanh\left( \frac{p_{12}e_1+p_{14}e_2}{\mu _{\chi _1}}\right) -\eta _{\chi _1}\Gamma _{\chi _1}{\chi }_1 \end{aligned}$$

where, $\eta _{\theta _1}$ and $\eta _{\chi _1}$ are positive design constants.

According to Lamma 4^35^, the following inequality holds

24 $$\begin{aligned} \begin{aligned} |&p_{12}e_1+p_{14}e_2|\chi ^{M}_1-(p_{12}e_1+p_{14}e_2)\chi ^M_1 tanh\left( \frac{p_{12}e_1+p_{14}e_2}{\mu _{\chi _1}}\right) \le 0.2785\mu _{\chi _1}\chi ^{M}_1 \end{aligned} \end{aligned}$$

and applying Young’s inequality, we have

25 $$\begin{aligned} \begin{aligned} p_{12}e_1e_3&\le \dfrac{1}{2}p^2_{12}e^2_1+\dfrac{1}{2}e^2_3,\\ p_{14}e_2e_3&\le \dfrac{1}{2}p^2_{14}e^2_2+\dfrac{1}{2}e^2_3,\\ \widetilde{\theta }^T_1\theta _1&=\widetilde{\theta }^T_1(\theta ^*_1-\widetilde{\theta }_1)\le \dfrac{1}{2}\theta ^{*T}_1\theta ^{*}_1-\dfrac{1}{2}\widetilde{\theta }^T_1\widetilde{\theta }_1, \\ \widetilde{\chi }^T_1{\chi }_1&=\widetilde{\chi }^T_1(\chi ^M_1-\widetilde{\chi }_1)\le \dfrac{1}{2}{\chi ^{M}_1}^T\chi ^{M}_1-\dfrac{1}{2}\widetilde{\chi }^T_1\widetilde{\chi }_1. \end{aligned} \end{aligned}$$

By (22–25), it follows that the dynamic of $\dot{V}_1$ becomes:

26 $$\begin{aligned} \dot{V}_1&\le -\dfrac{1}{2}q_{11}e^2_1-\dfrac{1}{2}q_{14}e^2_2+\dfrac{1}{2}p^2_{12}e^2_1+\dfrac{1}{2}p^2_{14}e^2_2+e^2_3+0.2785\mu _{\chi _1}\chi ^{M}_1+\widetilde{\theta }^T_1\eta _{\theta _1}{\theta }_1+\widetilde{\chi }^T_1\eta _{\chi _1}{\chi }_1 \nonumber \\&\le -\dfrac{1}{2}(q_{11}-p^2_{12})e^2_1-\dfrac{1}{2}(q_{14}-p^2_{14})e^2_2+e^2_3+0.2785\mu _{\chi _1}\chi ^{M}_1\nonumber \\&\quad +\dfrac{\eta _{\theta _1}}{2}\theta ^{*T}_1\theta ^*_1-\dfrac{\eta _{\theta _1}}{2}\widetilde{\theta }^T_1\widetilde{\theta }_1+\dfrac{\eta _{\chi _1}}{2}{\chi ^{M}_1}^T\chi ^{M}_1-\dfrac{\eta _{\chi _1}}{2}\widetilde{\chi }^T_1\widetilde{\chi }_1 \end{aligned}$$

*Step 2.*

Next, the second second-order subsystem described by $(e_3, e_4)$ in the system error dynamics Eq. (11) is considered. The error states $e_3$ and $e_4$ are rewritten as

27 $$\begin{aligned} \left\{ \begin{array}{l} \dot{e}_3=e_4 \\ \begin{aligned} \dot{e}_4=&{}f_2(x_1,x_2,x_3,x_4,\tau )+\overline{d}_2-\dot{x}_{4c}+k_3\varsigma _3+k_4\varsigma _4+u \end{aligned} \end{array} \right. \end{aligned}$$

The unknown function $f_2(x_1, x_2, x_3, x_4, \tau )$ is approximated by a RBF NN $\theta ^{*T}_2\varphi _2(x_1, x_2, x_3, x_4, \tau )$ consequently, given a compact set $\Omega _2\in {R^5}$, let $\theta ^{*}_2$ and $\varepsilon ^*_2$ be such that for any $(x_1, x_2, x_3, x_4, \tau )\in \Omega _2$

28 $$\begin{aligned} \begin{aligned} f_2(x_1, x_2, x_3, x_4, \tau )=\theta ^{*T}_2\varphi _2(x_1, x_2, x_3, x_4, \tau )+ \varepsilon ^*_2 \end{aligned} \end{aligned}$$

where, $\theta ^{*}_2$ is the ideal weights vector and its upper bound is $|\theta ^{*}_2|\le \theta ^{M}_2$. $\varepsilon ^*_2$ shows the approximation error and its upper bound is $|\varepsilon ^*_2|\le \varepsilon ^M_2$.

It can be obtained from Eqs. (27) and (28)

29 $$\begin{aligned} \begin{aligned} \dot{e}_4&=\theta ^{*T}_2\varphi _2(x_1, x_2, x_3, x_4, \tau )+\varepsilon ^*_2+\overline{d}_2-\dot{x}_{4c}+k_3\varsigma _3+k_4\varsigma _4+u \end{aligned} \end{aligned}$$

The control variable *u* is designed as

30 $$\begin{aligned} \begin{aligned} u&=-k_3(x_3-x_{3c})-\theta ^{T}_2\varphi _2(x_1, x_2, x_3, x_4, \tau )-k_4(x_4-x_{4c})-{\chi }_2tanh\left( \frac{p_{22}e_3+p_{44}e_4}{\mu _{\chi _2}}\right) +\dot{x}_{4c} \end{aligned} \end{aligned}$$

where, both $k_3$ and $k_4$ will be specified later, $\theta _2$ is the estimation of $\theta ^{*}_2$ and the estimation error is $\widetilde{\theta }_2=\theta ^{*}_2-\theta _2$. $\chi _2$ is the estimation of $\chi ^M_2=\varepsilon ^{M}_2+\overline{d}^M_2$ and the estimation error is $\widetilde{\chi }_2=\chi ^M_2-{\chi }_2$. $\mu _{\chi _2}, p_{22}$ and $p_{24}$ are positive design constants.

The dynamics fo error $e_4$ can be obtained from Eqs. (29) and (30).

31 $$\begin{aligned} \begin{aligned} \dot{e}_4&=\theta ^{*T}_2\varphi _2(x_1,x_2, x_3, x_4, \tau )+\varepsilon ^*_2+\overline{d}_2-\dot{x}_{4c}+k_3\varsigma _3+k_4\varsigma _4-k_3(x_3-x_{3c})-k_4(x_4-x_{4c})\\&\quad -\theta ^{T}_2\varphi _1(x_1, x_2, x_3, x_4, \tau )-{\chi }_2tanh\left( \frac{p_{22}e_3+p_{44}e_4}{\mu _{\chi _2}}\right) +\dot{x}_{4c} \\&=-k_3e_3-k_4e_4+\widetilde{\theta }^T_2\varphi _1(x_1, x_2, x_3, x_4, \tau )+\varepsilon ^*_2+\overline{d}_2-{\chi }_2tanh\left( \frac{p_{22}e_3+p_{44}e_4}{\mu _{\chi _2}}\right) \end{aligned} \end{aligned}$$

$k_3$ and $k_4$ are chosen such that

32 $$\begin{aligned} \ddot{\vartheta }+k_4\dot{\vartheta }+k_3\vartheta =0 \end{aligned}$$

is asymptotically stable at $(\vartheta ,\dot{\vartheta })=(0, 0)$.

For consistent expression with $e_{12}$, the second second-order subsystem errors $(e_3,e_4)$ dynamics can be written as

33 $$\begin{aligned} \left\{ \begin{array}{l} \dot{e}_3=e_4 \\ \begin{aligned} \dot{e}_4&{}=-k_3e_3-k_4e_4+\widetilde{\theta }^T_2\varphi _2(x_1,x_2, x_3, x_4, \tau )+\varepsilon ^*_2+\overline{d}_2-{\chi }_2tanh\left( \dfrac{p_{22}e_3+p_{44}e_4}{\mu _{\chi _2}}\right) \end{aligned} \end{array} \right. \end{aligned}$$

It can be written in matrix form

34 $$\begin{aligned} \begin{aligned} \dot{e}_{34}&=A_2{e}_{34}+B_2\left( \widetilde{\theta }^T_2\varphi _2(x_1,x_2, x_3, x_4, \tau )+\varepsilon ^*_2+\overline{d}_2\right) -B_2{\chi }_2tanh\left( \frac{p_{22}e_3+p_{44}e_4}{\mu _{\chi _2}}\right) \end{aligned} \end{aligned}$$

where, $$e_{34}=\left[ \begin{array}{l}e_3 \\ e_4\end{array}\right]$$, $$A_2=\begin{bmatrix} 0 &{} 1\\ -k_3 &{} -k_4\end{bmatrix}$$, $$B_2=\left[ \begin{array}{l}0 \\ 1\end{array}\right]$$.

Samely, two positive parameters $k_3$ and $k_4$ can be chosen to make sure that the following two positive definite symmetry matrices exist.

$$\begin{aligned} P_2=\begin{bmatrix}p_{21}&{}p_{22} \\ p_{22} &{}p_{24}\end{bmatrix},\quad Q_2=\begin{bmatrix}q_{21}&{}0 \\ 0 &{}q_{24}\end{bmatrix} \end{aligned}$$

so that

35 $$\begin{aligned} A^T_2P_2+P_2A_2=-Q_2 \end{aligned}$$

The Lyapunov function candidate is chosen as follows:

36 $$\begin{aligned} V_2=\dfrac{1}{2}e^T_{34}P_2e_{34}+ \dfrac{1}{2}\widetilde{\theta }^T_2\Gamma ^{-1}_{\theta _2}\widetilde{\theta }_2+ \dfrac{1}{2}\widetilde{\chi }^T_2\Gamma ^{-1}_{\chi _2}\widetilde{\chi }_2 \end{aligned}$$

where $\Gamma _{\theta _2}$ and $\Gamma _{\chi _2}$ are positive definite. Its time derivative $\dot{V}_2$ is given by

37 $$\begin{aligned} \dot{V}_2&=-\dfrac{1}{2}e^T_{34}Q_2e_{34}+e^T_{34}P_2B_2\left( \widetilde{\theta }^T_2\varphi _2(x_1,x_2, x_3, x_4, \tau )\right. \left. +\varepsilon ^*_2+\overline{d}_2-{\chi }_2tanh\left( \frac{p_{22}e_3+p_{24}e_4}{\mu _{\chi _2}}\right) \right) \nonumber \\&\quad +\widetilde{\theta }^T_2\Gamma ^{-1}_{\theta _2}\dot{\widetilde{\theta }}_2 +\widetilde{\chi }^T_2\Gamma ^{-1}_{\chi _2}\dot{\widetilde{\chi }}_2\nonumber \\&=-\dfrac{1}{2}q_{21}e^2_3-\dfrac{1}{2}q_{24}e^4_2+(p_{22}e_3+p_{24}e_4)(\varepsilon ^*_2+\overline{d}_2)-(p_{22}e_3+p_{24}e_4){\chi }_2tanh\left( \frac{p_{22}e_3+p_{24}e_4}{\mu _{\chi _2}}\right) \nonumber \\&\quad +\widetilde{\theta }^T_2\Gamma ^{-1}_{\theta _2}\Big ((p_{22}e_3+p_{24}e_4)\Gamma _{\theta _2}\varphi _2(x_1,x_2, x_3, x_4, \tau )\Big .\Big .-\dot{\theta }_4\Big )+\widetilde{\chi }^T_2\Gamma ^{-1}_{\chi _2}\dot{\widetilde{\chi }}_2\nonumber \\&\le -\dfrac{1}{2}q_{21}e^2_3-\dfrac{1}{2}q_{24}e^4_2+0.2785\mu _{\chi _2}\chi ^M_2+\widetilde{\theta }^T_2\Gamma ^{-1}_{\theta _2}\left( (p_{22}e_3+p_{24}e_4)\Gamma _{\theta _2}\varphi _2(x_1,x_2, x_3, x_4, \tau )-\dot{\theta }_2\right) \nonumber \\&\quad +\widetilde{\chi }^T_2\Gamma ^{-1}_{\chi _2}\bigg ((p_{22}e_3+p_{24}e_4)\Gamma _{\chi _2}tanh\left( \frac{p_{22}e_3+p_{24}e_4}{\mu _{\chi _2}}\right) -\dot{{\chi }}_2\bigg ) \end{aligned}$$

The parameter updating law $\dot{\theta }_4$ is designed as

38 $$\begin{aligned} \dot{\theta }_2= & {} (p_{22}e_3+p_{24}e_4)\Gamma _{\theta _2}\varphi _2(x_1,x_2, x_3, x_4, \tau )-\eta _{\theta _2}\Gamma _{\theta _2}\theta _2 \end{aligned}$$

39 $$\begin{aligned} \dot{{\chi }}_2= & {} (p_{22}e_3+p_{24}e_4)\Gamma _{\chi _2}tanh\left( \frac{p_{22}e_3+p_{24}e_4}{\mu _{\chi _2}}\right) -\eta _{\chi _2}\Gamma _{\chi _2}{\chi }_2 \end{aligned}$$

where, $\eta _{\theta _2}$ and $\eta _{\chi _2}$ are positive design constants. According to the complete square inequality

40 $$\begin{aligned} \begin{aligned}{}&\widetilde{\theta }^T_2\theta _2=\widetilde{\theta }^T_2(\theta ^*_2-\widetilde{\theta }_2)\le \dfrac{1}{2}\theta ^{*T}_2\theta ^{*}_2-\dfrac{1}{2}\widetilde{\theta }^T_2\widetilde{\theta }_2 \\&\widetilde{\chi }^T_2{\chi }_2=\widetilde{\chi }^T_2(\chi ^M_2-\widetilde{\chi }_2)\le \dfrac{1}{2}{\chi ^{M}_2}^T\varepsilon ^{M}_2-\dfrac{1}{2}\widetilde{\chi }^T_2\widetilde{\chi }_2 \end{aligned} \end{aligned}$$

Substituting Eqs. (38–40) into Eq. (37), it follows that the dynamic of $\dot{V}_2$ becomes:

41 $$\begin{aligned} \begin{aligned} \dot{V}_2&\le -\dfrac{1}{2}q_{21}e^2_3-\dfrac{1}{2}q_{24}e^2_4+0.2785\mu _{\chi _2}\chi ^M_2-\dfrac{\eta _{\theta _2}}{2}\widetilde{\theta }^T_2\widetilde{\theta }_2+\dfrac{\eta _{\theta _2}}{2}\theta ^{*T}_2\theta ^*_2-\dfrac{\eta _{\chi _2}}{2}\widetilde{\chi }^T_2\widetilde{\chi }_2+\dfrac{\eta _{\chi _2}}{2}{\chi ^{M}_2}^T\chi ^M_2 \end{aligned} \end{aligned}$$

**Figure 1:**
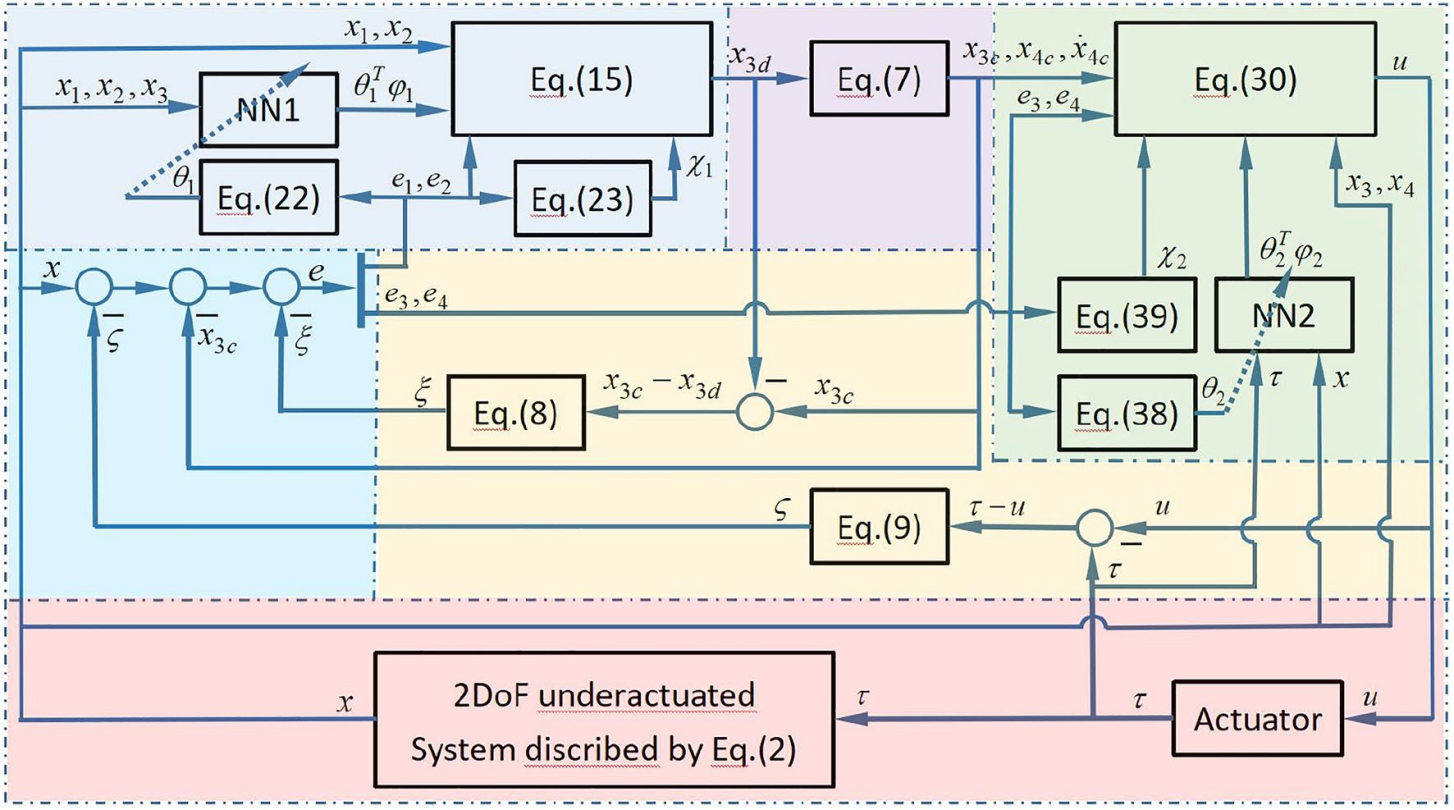
The architecture of the control scheme.

The architecture of the control scheme is shown in Fig.1. From left to right and from top to bottom, the functional zones with different backgrounds are as follows: first step of backstepping, second-order filter, second step of backstepping, error systems, auxiliary systems, and the contolled 2DoF underactuated system.

#### Remark 5

In the second-order backstepping design process, traditional linear system design ideas were borrowed, but the virtual controller Eq. (15) and controller Eq. (30) are not linearly designed for the first subsystem and the second subsystem. The last two terms on the right side of Eqs. (19) and (34) are functions of $x_i$ or $e_i$, $i=1,2$, which also makes it impossible to analyze the stability of the system using linear system theory. Ultimately, the stability of the system is proved using Lyapunov stability theory in the next subsection. The advantage of this design approach is that the system dynamics can mainly be determined by linear systems even in the approximation process of the neural networks and adaptive laws, so the control system performance can be improved according to the idea of linear systems.

#### Remark 6

Taking full account of the cascade model structure of the 2DoF underactuated systems Eq. (3) and the structure of error dynamics Eq. (11), every backstepping step is designed for a second-order subsystem. Compared with first-order backstepping^9,21,22,24^, the proposed second-order backstepping has the following advantages: firstly, the number of backstepping design steps is the same as the number of system DoFs, which can reduce the number of design steps and simplify the design process; secondly, the dynamic performance of the each subsystem can be adjusted by changing parameters ($k_1, k_2$ for the first subsystem and $k_3, k_4$ for the second subsystem) according to the idea of classic linear system control theory.

#### Remark 7

Considering that each backstepping step is designed for a second-order system, the second-order derivative of the virtual control is needed to accomplish the design process. Therefore, a second-order filter is integrated into the second backstepping step to replace the second-order derivative, which cannot be achieved by a first-order filter^21–23,36–38^. Moreover, an error dynamic auxiliary system is designed for the second-order filter to compensate for filtering errors and improve the control performance.

#### Remark 8

The control algorithm is proposed for a class of underactuated systems that has two DoFs with configuration vector $q=(q_1, q_2) ^ T$, and its positive define symmetric inertia matrix only depends on $q_2$, i.e. $M=M (q_2)$. In fact, the proposed algorithm is applicable to pure feedback as shown in Eq. (4) or strict feedback systems. That is to say, The proposed backstepping design process is applicable to all underactuated systems that can be transformed into the same structure of Eq. (4) through a global coordinate change, and can also be extended to high-order pure feedback or strict feedback systems with the structure as Eq.(4).

### Stability analysis

The nonlinear controller has been designed and the stability analysis will be accomplished for the class of underactuated systems in this subsection.

#### Theorem 1

Consider the 2DoF underactuated systems described by Eq. (2) with input saturation Eq. (6) and uncertain disturbances, which can be transformed into the cascade model in feedback from Eq. (4) through the global change of coordinates Eq. (3). For any bounded initial conditions, the nonlinear controller Eq. (30) and the virtual controller Eq. (15) with second-order filter Eq. (7), the NN weight update laws Eqs. (22, 38), the adaptive laws Eqs. (23, 39) and auxiliary systems Eqs. (8, 9) for filtering error and input saturation, can ensure that the system dynamics converge into a small neighborhood of the origin and all variables of the closed-loop control systems remain bounded.

#### Proof

Consider the Lyapunov function candidate

42 $$\begin{aligned} \begin{aligned} V&=V_1+V_2\\&=\dfrac{1}{2}e^T_{12}P_2e_{12}+ \dfrac{1}{2}\widetilde{\theta }^T_1\Gamma ^{-1}_{\theta _1}\widetilde{\theta }_1+ \dfrac{1}{2}\widetilde{\chi }^T_1\Gamma ^{-1}_{\chi _1}\widetilde{\chi }_1+\dfrac{1}{2}e^T_{34}P_2e_{34}+ \dfrac{1}{2}\widetilde{\theta }^T_2\Gamma ^{-1}_{\theta _2}\widetilde{\theta }_2+ \dfrac{1}{2}\widetilde{\chi }^T_2\Gamma ^{-1}_{\chi _2}\widetilde{\chi }_2 \end{aligned} \end{aligned}$$

It is noted that

$$\begin{aligned} \theta ^{*}_1\theta ^{*T}_1\le {\theta ^M_1}^T\theta ^{M}_1, \quad \theta ^{*}_2\theta ^{*T}_2\le {\theta ^M_2}^T\theta ^{M}_2. \end{aligned}$$

According to Eqs. (26) and (41), we have

$$\begin{aligned} \dot{V}&\le -\dfrac{1}{2}(q_{11}-p^2_{12})e^2_1-\dfrac{1}{2}(q_{14}-p^2_{14})e^2_2+e^2_3+0.2785\mu _{\chi _1}\chi ^{M}_1+\dfrac{\eta _{\theta _1}}{2}\theta ^{*T}_2\theta ^*_2-\dfrac{\eta _{\theta _1}}{2}\widetilde{\theta }^T_1\widetilde{\theta }_1\\&\quad +\dfrac{\eta _{\chi _1}}{2}{\chi ^{M}_1}^T\chi ^{M}_1-\dfrac{\eta _{\chi _1}}{2}\widetilde{\chi }^T_1\widetilde{\chi }_1\\&\quad -\dfrac{1}{2}q_{21}e^2_3-\dfrac{1}{2}q_{24}e^2_4+0.2785\mu _{\chi _2}\chi ^M_2-\dfrac{\eta _{\theta _2}}{2}\widetilde{\theta }^T_2\widetilde{\theta }_2+\dfrac{\eta _{\theta _2}}{2}\theta ^{*T}_2\theta ^*_2\\&\quad -\dfrac{\eta _{\chi _2}}{2}\widetilde{\chi }^T_2\widetilde{\chi }_2+\dfrac{\eta _{\chi _2}}{2}{\chi ^M_2}^T\chi ^M_2\\&\le -\gamma V+\left( -\dfrac{1}{2}(q_{11}-p^2_{12})e^2_1-\dfrac{1}{2}(q_{14}-p^2_{14})e^2_2-\dfrac{1}{2}(q_{21}-2)e^2_3-\dfrac{1}{2}q_{24}e^2_4\right. \\&\left. \quad +\dfrac{1}{2}\gamma e^T_{12}P_1e_{12}+\dfrac{1}{2}\gamma e^T_{34}P_2e_{34}\right) \\&+\left( \dfrac{1}{2}\gamma \widetilde{\theta }^T_1\Gamma ^{-1}_{\theta _1}\widetilde{\theta }_1-\dfrac{\eta _{\theta _1}}{2}\widetilde{\theta }^T_2\widetilde{\theta }_2+\dfrac{1}{2}\gamma \widetilde{\theta }^T_2\Gamma ^{-1}_{\theta _2}\widetilde{\theta }_2-\dfrac{\eta _{\theta _2}}{2}\widetilde{\theta }^T_2\widetilde{\theta }_2\right. \\&\left. \quad +\dfrac{1}{2}\gamma \widetilde{\chi }^T_1\Gamma ^{-1}_{\chi _1}\widetilde{\chi }_1-\dfrac{\eta _{\chi _1}}{2}\widetilde{\chi }^T_1\widetilde{\chi }_1+\dfrac{1}{2}\gamma \widetilde{\chi }^T_2\Gamma ^{-1}_{\chi _2}\widetilde{\chi }_2-\dfrac{\eta _{\chi _2}}{2}\widetilde{\chi }^T_2\widetilde{\chi }_2 \right) \\&\quad +\left( 0.2785\mu _{\chi _1}\chi ^{M}_1+0.2785\mu _{\chi _2}\chi ^M_2+\dfrac{\eta _{\theta _1}}{2}{\theta ^M_1}^T\theta ^M_1\right. \\&\left. \quad +\dfrac{\eta _{\chi _1}}{2}{\chi ^{M}_1}^T\chi ^{M}_1+\dfrac{\eta _{\theta _2}}{2}{\theta ^M_2}^T\theta ^M_2+\dfrac{\eta _{\chi _2}}{2}{\chi ^{M}_2}^T\chi ^{M}_2\right) \end{aligned}$$

According to Young’s inequality, we have

43 $$\begin{aligned} \begin{aligned} \dfrac{1}{2}\gamma e^T_{12}P_1e_{12}&=\dfrac{1}{2}\gamma (p_{11}e^2_1+2p_{12}e_1e_2+p_{14}e^2_2)\\&\le \dfrac{1}{2}\gamma (p_{11}+p^2_{12})e^2_1+\dfrac{1}{2}\gamma (p_{14}+1)e^2_2\\ \dfrac{1}{2}\gamma e^T_{34}P_2e_{34}&=\dfrac{1}{2}\gamma (p_{21}e^2_3+2p_{22}e_3e_4+p_{24}e^2_4)\\&\le \dfrac{1}{2}\gamma (p_{21}+p^2_{22})e^2_3+\dfrac{1}{2}\gamma (p_{24}+1)e^2_4 \end{aligned} \end{aligned}$$

Therefore,

44 $$\begin{aligned} \dot{V}&\le -\gamma V+\dfrac{1}{2}\left( p^2_{12}+\gamma (p_{11}+p^2_{12})-q_{11}\right) e^2_1 +\dfrac{1}{2}\left( p^2_{14}+\gamma (p_{14}+1)-q_{12}\right) e^2_2\nonumber \\&\quad +\dfrac{1}{2}\left( 2+\gamma (p_{21}+p^2_{22})-q_{21}\right) e^2_3\nonumber \\&\quad +\dfrac{1}{2}\left( \gamma (p_{24}+1)-q_{24}\right) e^2_4+\dfrac{1}{2}\widetilde{\theta }^T_1\left( \gamma \Gamma ^{-1}_{\theta _1}-\eta _{\theta _1}I\right) \widetilde{\theta }_1\nonumber \\&\quad +\dfrac{1}{2}\widetilde{\theta }^T_2\left( \gamma \Gamma ^{-1}_{\theta _2}-\eta _{\theta _2}I\right) \widetilde{\theta }_2+\dfrac{1}{2}\widetilde{\chi }^T_1\left( \gamma \Gamma ^{-1}_{\chi _1}-\eta _{\chi _1}I\right) \widetilde{\chi }_1\nonumber \\&\quad +\dfrac{1}{2}\widetilde{\chi }^T_2\left( \gamma \Gamma ^{-1}_{\chi _2}-\eta _{\chi _2}I\right) \widetilde{\chi }_2+C \nonumber \\&\le -\gamma V+C \end{aligned}$$

where,

$$\begin{aligned} \begin{array}{l} \begin{aligned} \gamma :&{}\le min\bigg (\dfrac{q_{11}-p^2_{12}}{p_{11}+p^2_{12}}, \dfrac{q_{12}-p^2_{14}}{p_{14}+1},\dfrac{q_{21}-2}{p_{21}+p^2_{22}}, \dfrac{q_{24}}{p_{24}+1}, \eta _{\theta _1}\lambda _{min}(\Gamma _{\theta _1}),\eta _{\theta _2}\lambda _{min}(\Gamma _{\theta _2}),\eta _{{\chi _1}}\lambda _{min}(\Gamma _{{\chi _1}}),\eta _{{\chi _2}}\lambda _{min}(\Gamma _{{\chi _2}})\Bigg )\\ C&{}:=0.2785\mu _{\chi _1}\chi ^{M}_1+0.2785\mu _{\chi _2}\chi ^M_2+\dfrac{\eta _{\theta _1}}{2}{\theta ^M_1}^T\theta ^M_1+\dfrac{\eta _{\chi _1}}{2}{\chi ^{M}_1}^T\chi ^{M}_1+\dfrac{\eta _{\theta _2}}{2}{\theta ^M_2}^T\theta ^M_2+\dfrac{\eta _{\chi _2}}{2}{\chi ^{M}_2}^T\chi ^{M}_2 \end{aligned} \end{array} \end{aligned}$$

When the corresponding positive design parameters $k_1$, $k_2$ , $k_3$, $k_4$, $\eta _{\theta _1}$, $\eta _{\theta _2}$, $\eta _{\chi _1}$, $\eta _{\chi _2}$, $\mu _{\chi _1}$, $\mu _{\chi _2}$ and if we choose the positive definite symmetry matrices $P_1$, $P_2$, $Q_1$, $Q_2$, $\Gamma _{\theta _1}$, $\Gamma _{\theta _2}$, $\Gamma _{\chi _1}$, $\Gamma _{\chi _2}$ to meet the following inequalities:

$$\begin{aligned} q_{11}-p^2_{12}>0,\quad q_{12}-p^2_{14}>0,\quad q_{21}-2>0. \end{aligned}$$

Equation (44) can be solved as

$$\begin{aligned} 0\le V(t)\le \dfrac{C}{\gamma }+\left( V(0)-\dfrac{C}{\gamma }\right) e^{-\gamma t} \end{aligned}$$

therefore

$$\begin{aligned} \underset{t\rightarrow \infty }{lim}V(t)\le \dfrac{C}{\gamma } \end{aligned}$$

Thus, $e_1$, $e_2$, $e_3$ and $e_4$ remain bounded and converge into a small neighborhood of the origin. From the error dynamics, auxiliary dynamics and the backstepping process, it can be seen that all other variables $x_1$, $x_2$, $x_3$, $x_4$, $\varsigma _1$, $\varsigma _2$, $\varsigma _3$, $\varsigma _4$, $\xi _1$ and $\xi _2$ are bounded and converge into a small neighborhood of the origin. This concludes the proof. $\square$

The improved global change of coordinates Eq. (3) is invertible. According to the invertible transformation Eq. (5), $(x_1, \dot{x}_1, x_2, \dot{x}_2)$ converges into a small neighborhood of origin (0, 0, 0, 0) can deduce that $(q_1, \dot{q}_1, q_2, \dot{q}_2)$ converges into a small neighborhood of origin (0, 0, 0, 0).

In the second-order backstepping design process, adaptive techniques are used to estimate the sum of the upper bound of the NN approximation error and the upper bound of the disturbance. This estimated value is also applied in the controller to improve the control performance. From the assumption 1, the maximum value of the disturbance is limited, and according to the universal approximation theorem, the approximation error of neural networks is also bounded, so the sum of the above two is also bounded. The stability proof process have proved that as long as the maximum value exists, the system is stable according to Eq. (44).

## Numerical simulations

The proposed control algorithm is simulated and validated using two 2DoF underactuated systems: the TORA benchmark system and the IWP benchmark system. A simulation testbed is built in the Matlab/Simulink environment, running under Windows 10.

### TORA and IWP dynamics

#### TORA

**Figure 2:**
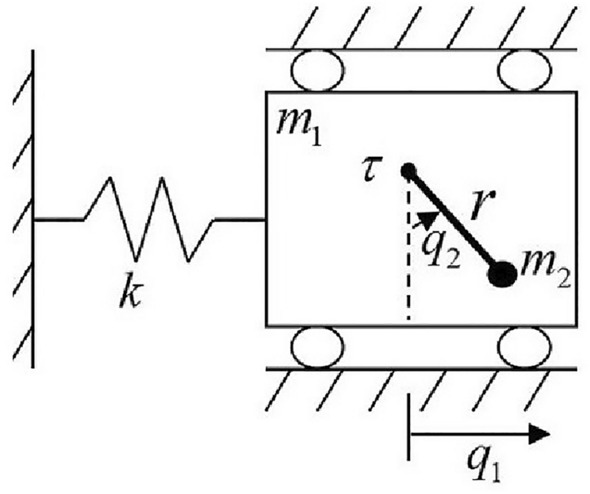
TORA system configuration.

The TORA benchmark system is shown in Fig.2. A linear spring of stiffness *k* is fixed on a wall on one end and connected with a cart of mass $m_1$ on the other end. The cart can only move in one dimension and its position is $q_1$. An active rotational actuator driven by a motor is equipped in the cart. The parameter $m_2$ represents the equivalent mass of rotational actuator, parameter *r* represents the rotate radius, parameter *I* represents the moment of inertia, and parameter $q_2$ represents the rotate angle. The fact that the cart is not equipped with a motor makes the TORA a benchmark underactuated system: $\tau$ is the only control input while $q_1$ and $q_2$ are the two DoF variables. Its theoretical dynamics can be described as follows.

**Figure 3:**
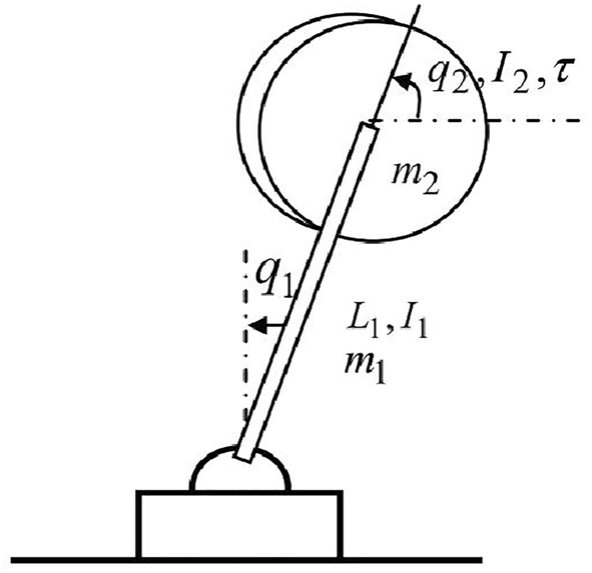
IWP system configuration.

45 $$\begin{aligned} \left\{ \begin{array}{l} (m_1+m_2)\ddot{q}_1+m_{2}rcos(q_2)\ddot{q}_2-m_{2}rsin(q_2)\dot{q}_2^2+kq_1=d_1 \\ m_{2}rcos(q_2)\ddot{q}_1+(m_2r^2+I)\ddot{q}_2+m_2grsin(q_2)=\tau +d_2 \end{array} \right. \end{aligned}$$

Equation (3) in Sect. 2 can transform the above system dynamics Eq. (45) into the form as Eq. (4), where

$$\begin{aligned} f_1(x_1,x_3)&=-\dfrac{kx_1}{m_1+m_2}+\dfrac{km_2r}{(m_1+m_2)^2}sinx_3-x_3,\\ \overline{d}_1&=\Delta {f}_2(x_1,x_3)+(g_{d1}+\Delta {g_{d1}})d_1,\\ g_{d1}&=(m_1+m_2)^{-1},\\ f_2(x_1, x_3,x_4,\tau )&=g_4(x_3)\bigg (km_2r\left( x_1-\dfrac{m_2rsin{x_3}}{m_1+m_2}\right) cos{x_3}-(m_2r)^2sin{x_3}cos{x_3}x_4^2-(m_1+m_2)m_2grsin{x_3}\bigg )\\&\quad +\left( (m_1+m_2)g_4(x_3)-1\right) \tau , \\ g_4(x_3)&=\left( (m_1+m_2)(m_2r^2+I)-(m_2rcosx_3)^2\right) ^{-1},\\ \overline{d}_2&=\Delta {f}_4(x_1,x_3,x_4,\tau )+\left( {g}_{d2}(x_3)+\Delta {g}_{d2}(x_3)\right) d_2+\left( g_{d3}(x_3)+\Delta {g}_{d3}(x_3)\right) d_1,\\ g_{d2}(x_3)&=g_4(x_3)(m_1+m_2), \quad g_{d3}(x_3)=-g_4(x_3)m_2rcos{x_3}. \end{aligned}$$

#### IWP

The IWP benchmark system is shown in Fig. 3. A physical pendulum of mass $m_1$ is fixed on the ground on one end and the other end is a revolving wheel of equivalent mass $m_2$. The torque $\tau$ outputted from the motor can make the revolving wheel rotate and generate a coupling dynamic for the physical pendulum. The actuated revolving wheel and the unactuated pendulum make the IWP system a benchmark underactuated system: $\tau$ is the only control input while $q_1$ and $q_2$ are the two DoF variables. Its theoretical dynamics can be described as follows.

46 $$\begin{aligned} \left\{ \begin{array}{l} m_{11}\ddot{q}_1+m_{12}\ddot{q}_2-m_{0}gsin(q_1)=d_1 \\ m_{21}\ddot{q}_1+m_{22}\ddot{q}_2=\tau +d_2 \end{array} \right. \end{aligned}$$

Equation (3) can transform the above system dynamics Eq. (46) into the form as Eq. (4), where

$$\begin{aligned} f_1(x_1,x_3)&=\dfrac{m_0gsin\left( x_1-\dfrac{m_{12}x_3}{m_{11}}\right) }{m_{11}},\quad \overline{d}_1=\Delta {f}_2(x_1,x_3)+(g_{d1}+\Delta {g_{d1}})d_1,\quad g_{d1}=m^{-1}_{11},\\ f_2(x_1, x_3, x_4,\tau )&=-g_4m_{21}m_0gsin\left( x_1-\dfrac{m_{12}x_3}{m_{11}}\right) +\left( g_4m_{11}-1\right) \tau ,\quad g_4=\left( m_{11}m_{22}-m_{12}m_{21}\right) ^{-1}\\ \overline{d}_2&=\Delta {f}_4(x_1,x_3,x_4,\tau )+\left( {g}_{d2}(x_3)+\Delta {g}_{d2}(x_3)\right) d_2+\left( g_{d3}(x_3)+\Delta {g}_{d3}(x_3)\right) d_1, \\ g_{d2}&=g_4m_{11},\quad g_{d3}=-g_4m_{21} \end{aligned}$$

### Friction model and external input disturbance

In the practical systems, there exist frictions for each DoF in the two benchmark systems. In this section, the following friction model^39^ is chosen for both TORA and IWP to treat the static Coulomb model and viscous friction:

47 $$\begin{aligned} F_i=\sigma _{oi}\dot{q}_i+F_{ci}sign(\dot{q}_i), i=1,2 \end{aligned}$$

where, $\sigma _{oi}>0$ is the viscous friction coefficient and $F_{ci}>0$ is the Coulomb friction coefficient, corresponding for the *i*-th DoF. The signum function *sign*(*q*) is is defined by

48 $$\begin{aligned} sign({q})=\left\{ \begin{array}{l} 1 \quad \quad {q}>0 \\ 0 \quad \quad {q}=0 \\ -1 \ \quad {q}<0 \\ \end{array} \right. \end{aligned}$$

Moreover, the same external input disturbances^28^ acting on the actuated DoF for both the TORA and the IWP are set to be

49 $$\begin{aligned} d_{2n}(t)=\dfrac{10sin(t)}{10+0.5t^2} \end{aligned}$$

Therefore, the input disturbances $d_1$ and $d_2$ in Eq. (2) are

50 $$\begin{aligned} d_1&=-\sigma _{o1}\dot{q}_1-F_{c1}sign(\dot{q}_1) \end{aligned}$$

51 $$\begin{aligned} d_2&=\dfrac{10sin(t)}{10+0.5t^2}-\sigma _{o2}\dot{q}_2-F_{c2}sign(\dot{q}_2) \end{aligned}$$

where, $\sigma _{o1}=\sigma _{o2}=0.1$ and $F_{c1}=F_{c1}=0.2$ are used in the simulations.

### Simulation parameters

In the simulations, the system parameters^12^ and initial states are listed in Table 1, in which some internal uncertainties are included through changing some system parameter from $\text {SP}_{{\text {TORA1}}}$ to $\text {SP}_{{\text {TORA2}}}$ and from $\text {SP}_{{\text {IWP1}}}$ to $\text {SP}_{{\text {IWP2}}}$. The control parameters are listed in Table 2 that are chosen for both the TORA system and the IWP system.

**Table 1:** The system parameters and initial state in the simulation.

Name	Abbreviation	Parameter value
System parameters of TORA	$\text {SP}_{{\text {TORA1}}}$	$m_1=10kg, \quad m_2=1kg,\quad k=5N/m,\quad r=1m, \quad I=1kg\cdot m^2$
	$\text {SP}_{{\text {TORA2}}}$	$m_1=12kg(+20\%\text {error}),\quad m_2=1.2kg(+20\%\text {error}),$ $k=5N/m,\quad r=1m, \quad I=1kg\cdot m^2$
System parameters of IWP	$\text {SP}_{{\text {IWP1}}}$	$m_0=0.191kg\cdot m,\quad m_{11}=0.0543kg\cdot m^2,$ $m_{12}=m_{21}=m_{22}=0.0027kg\cdot m^2$
	$\text {SP}_{{\text {IWP2}}}$	$m_0=0.2292kgm(+20\%\ \text {error}),\quad m_{11}=0.06516kgm^2(+20\%\ \text {error}),$ $m_{12}=m_{21}=m_{22}=0.00324kgm^2(+20\%\ \text {error})$
Initial states for TORA	$\text {IS}_{{\text {TORA1}}}$	$(x_1,x_2,x_3,x_4)=(0.01,0,\pi /6,0)$ $\text {i.e.}\quad (q_1,\dot{q}_1,q_2,\dot{q}_2)=(-35.5\text {mm},0\text {mm/s},30\text {deg},0\text {deg/s})$
	$\text {IS}_{{\text {TORA2}}}$	$(x_1,x_2,x_3,x_4)=(0,0,-\pi /6,0)$ $\text {i.e.}\quad (q_1,\dot{q}_1,q_2,\dot{q}_2)=(45.45\text {mm},0\text {mm/s},-30\text {deg},0\text {deg/s})$
Initial state for IWP	$\text {IS}_{{\text {IWP}}}$	$(x_1,x_2,x_3,x_4)=(\pi /6,0,0,0)$ $\text {i.e.}\quad (30\text {deg},0\text {deg/s},0\text {deg},0\text {deg/s})$

**Table 2:** The controller parameters in the simulation.

$$k_1=k_3=100,\quad k_2=k_4=50,\quad \eta _{\theta _1}=\eta _{\theta _2}=\eta _{\chi _1}=\eta _{\chi _2}=0.2,\quad \zeta =0.5,\quad \omega _n=20,\quad u_{max}=3,$$	
$$\quad u_{min}=-3, \quad \Gamma _{\theta _1}=\Gamma _{\theta _2}=diag([10,10,10,10,10,10,10]), \quad \Gamma _{\chi _1}=\Gamma _{\chi _2}=1, \quad \mu _{\chi _1}=\mu _{\chi _2}=0.5,$$	
$$P_1=P_2=\begin{bmatrix}98&{}1.5 \\ 1.5&{}0.23\end{bmatrix},\quad Q_1=Q_2=\begin{bmatrix}300&{}0 \\ 0 &{}20\end{bmatrix}, \quad A_1=A_2=\begin{bmatrix}0&{}1 \\ -100 &{}-50\end{bmatrix}.$$	

### Simulation results and analysis

Two simulation studies are performed with TORA to demonstrate the performance of the closed-loop control system under different system parameters and initial states.

**Figure 4:**
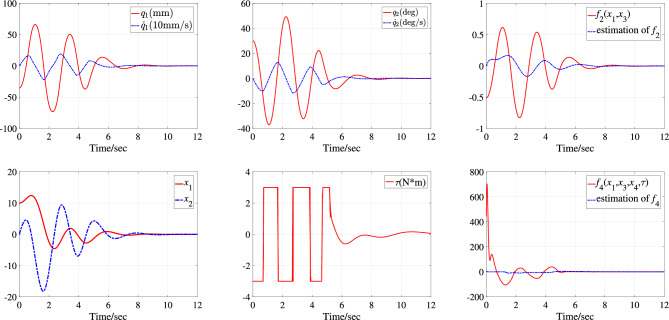
Simulation results with system parameters $\text {SP}_{\text {TORA1}}$ under initial state $\text {IS}_{\text {TORA1}}$.

The first simulation is performed with the system parameters $\text {SP}_{\text {TORA1}}$ under the initial state $\text {IS}_{\text {TORA1}}$. The simulation results, including the time-domain response of all system states, control input, and neural network approximations, are depicted in Fig. 4. It can be seen that under the action of the proposed controller, the active rotary actuator can stabilize the entire system after three oscillation cycles, and all system states converge to equilibrium state in about 9 seconds. During the process, the actuator enters saturation six times, and the system is still stable with the saturation compensation. In order to converge stably as soon as possible, the actuator needs to respond quickly and have a large output.

**Figure 5:**
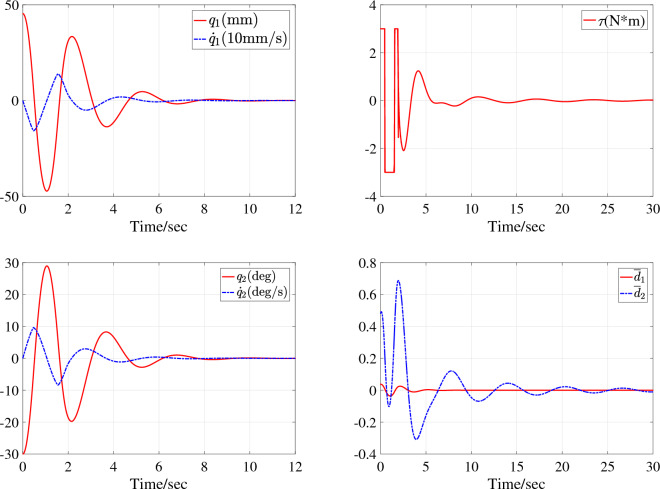
Simulation results with system parameters $\text {SP}_{\text {TORA2}}$ under initial state $\text {IS}_{\text {TORA2}}$.

The second simulation is performed with the system parameters $\text {SP}_{\text {TORA2}}$ under the initial state $\text {IS}_{\text {TORA2}}$ and the time-domain response of all system states, control input and the equivalent external disturbances after global coordinate changes are shown in Fig. 5. It can be seen that the active rotary actuator can stabilize the entire system after three oscillation cycles, all system states converge to equilibrium state in approximately 9 seconds, and the actuator enters saturation three times. Figures. 4 and 5 show that the second-order underactuated TORA system canbe stabilized to the equilibrium state of the system $(q_1,\dot{q_1},q_2,\dot{q_2})=(0,0,0,0)$ under different system parameters and initial states with the proposed second-order backstepping control algorithm.

**Figure 6:**
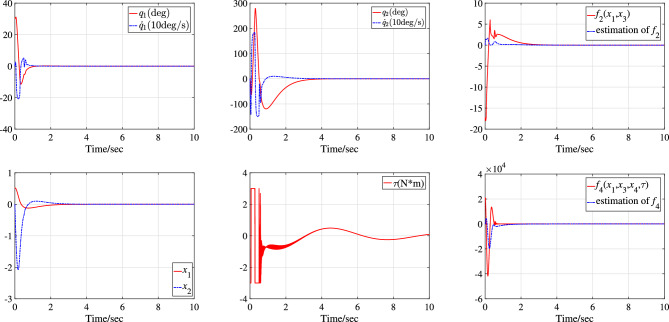
Simulation results with system parameters $\text {SP}_{\text {IWP1}}$ under initial state $\text {IS}_{\text {IWP}}$.

**Figure 7:**
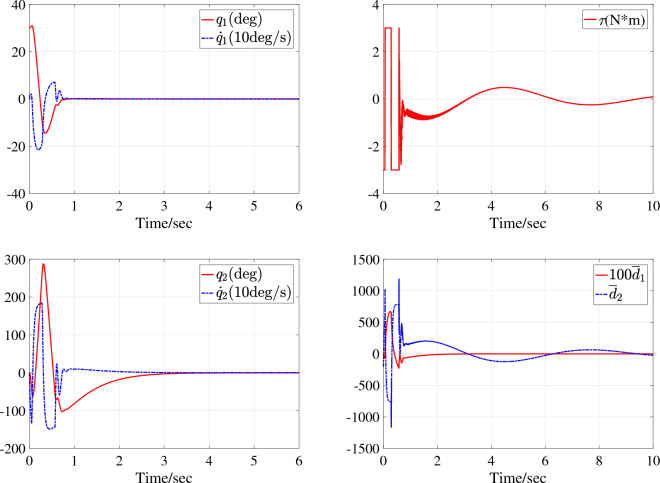
Simulation results with system parameters $\text {SP}_{\text {IWP2}}$ under initial state $\text {IS}_{\text {IWP}}$.

For the underactuated IWP system, two simulation studies are performed under initial state $\text {IS}_{\text {IWP}}$ and with different system parameters $\text {SP}_{\text {IWP1}}$ and $\text {SP}_{\text {IWP2}}$. The simulation results are corresponding depicted in Fig. 6 with system parameters $\text {SP}_{\text {IWP1}}$ under initial state $\text {IS}_{\text {IWP}}$ and Fig. 7 with system parameters $\text {SP}_{\text {IWP2}}$ under initial state $\text {IS}_{\text {IWP}}$. In Fig. 6, the motor assembled on the revolving wheel stabilizes the physical pendulum in about 3s and the system states $x_1$ and $x_2$ converge to the equilibrium state in one oscillation period. The control action $\tau$ enters saturation three times when the system states are far from equilibrium state and continues to be used to suppress system disturbances and internal uncertainties when the system states are in equilibrium state. In Fig. 7, the equivalent disturbances $\overline{d}_1$ and $\overline{d}_2$ are plotted and the control process is similar to the Fig. 6. In sum, the actuator has a large gain to stabilize the system, all the system states are stabilized to the equilibrium state of the system $(q_1,\dot{q_1},q_2,\dot{q_2})=(0,0,0,0)$ in about 4s and then the control input is only used to suppress external interference.

In Figs. 4 and 6, the NN can asymptotically approximate its function and the reason why the NN does not approximate its function very quickly is that the system state and uncertain disturbances change rapidly at the beginning of control and the parameter adaptation rates designed in this paper are for the purpose of system stability but not only for the approximation.

The reason that the control input does not converge to zero in Figs .4, 5, 6, 7 is that the disturbances persist throughout the simulation process. Figures 5 and 7 give the dynamics of the equivalent external disturbances acting on each DoF. The results show that the control action will eventually converge to zero only after the disturbance disappears. Moreover, according to the expressions of $\overline{d}_1$ and $\overline{d}_2$, the equivalent uncertain disturbances are different for TORA and IWP with the same parameter change rate and external disturbances even if both $d_1$ and $d_2$ are same for TORA and IWP.

In short, from the above simulation results, the proposed control method can make the 2DoF underactuated TORA benchmark system and IWP benchmark system converge to their equilibrium position under different system parameters, different initial states, uncertain disturbances, and input saturation.

#### Remark 9

From Eq. (9), it can be seen that the auxiliary system only works when the actuator is in a saturated state. For a system that prioritizes stability, a large actuator gain is necessary. A novel idea^40^ to handle actuator saturation has been proposed, in which a new saturation function is effectively combined with BLF-based control design to prevent the high control gains that lead to the large control amplitudes and as result reduces the risk of actuators saturation. Further research is needed to determine whether this idea is suitable for underactuated systems that prioritize stability. Moreover, from Eqs. (15) and (30), only the upper bound of the sum of NN approximation error and uncertain disturbance is needed in the virtual controller and actual controller. If the complete information of disturbances can be obtained for controller design, it will inevitably improve the system performance and so nonlinear disturbance observation^29,41^ is the next attempt.

#### Remark 10

The system control performance can be optimized by adjusting the design parameters used in the two backstepping steps as stated in Remark 5. According to linear system theory, the general strategy to select controller parameters (taking Step 1 as an example) is: firstly to determine $k_1$ and $k_2$ (i.e. matrix $A_1$) from the expected eigenvalues of Eq. (18); then determine matrix $Q_1$ from the control weights of $x_1$ and $x_2$; finally to obtain a positive definite matrix $P_1$ through solving the Eq. (20). If $q_{11}-p^2_{12}>0,\quad q_{12}-p^2_{14}>0,\quad q_{21}-2>0$ hold, the system is stable. Other parameters ( $\eta _{\theta _1}$, $\eta _{\theta _2}$, $\eta _{\chi _1}$, $\eta _{\chi _2}$, $\mu _{\chi _1}$, $\mu _{\chi _2}$, $\Gamma _{\theta _1}$, $\Gamma _{\theta _2}$, $\Gamma _{\chi _1}$, $\Gamma _{\chi _2}$ ) directly determine the convergence speed of their corresponding neural networks or adaptive laws. The reason for using the same design parameters in the two steps for TORA and IWP is that our main objective is to research the proposed adaptive second-order backstepping control design scheme for the 2DoF underactuated systems, and to ensure that the closed-loop system is stable and feasible. Moreover, no literature has been found about the aforementioned class of 2DoF underactuated systems with inertial parameter uncertainties, external disturbances, and input saturation, therefore the superiority of the proposed algorithm has not been studied from a comparative research.

#### Remark 11

A lot of simulations have been performed and the results show that the proposed algorithm can stabilize the system to its zero state, i.e. $(q_1, \dot{q}_1, q_2, \dot{q}_2)=(0, 0, 0, 0)$, but a feasible Lyapunov function can not be found for the 2DoF underactuated systems so as to support the asymptotic stability. This is the reason to prove the system approaches to a small neighborhood of (0, 0, 0, 0) and all variables of the closed-loop control system are bounded in the Sect. 3.

## Conclusions

The 2DoF underactuated system dynamics is transformed into a nonlinear cascade model in the feedback form. An adaptive second-order backstepping control design procedure is proposed considering the underactuated system as a pure feedback system to obtain a nonlinear control algorithm. The input saturation and uncertain disturbances are considered in the design process. A second-order filter is applied to replace the second-order derivatives of the virtual control so as to overcome the EoC problem. A filtering error auxiliary system is designed. Taking full account of the cascade feedback structure of the 2DoF underactuated systems, each backstepping step is designed for a second-order subsystem. Thus, the number of system DoFs is the same as the number of backstepping steps, which reduces the number of design steps and simplifies the design process. The closed-loop stability of the 2DoF underactuated systems is analyzed with the Lyapunov stability theory. The control performance is verified by numerical simulations.

## Data Availability

Te datasets used and/or analyzed during the current study are available from the corresponding author on reasonable request.

## References

[1] Spong, M. W. Underactuated mechanical systems. In Control problems in robotics and automation, 135–150 (Springer, 1998). 10.1007/BFb0015081.

[2] Olfati-Saber, R. Nonlinear control of underactuated mechanical systems with application to robotics and aerospace vehicles. Ph.D. thesis, Massachusetts Institute of Technology (2001). https://dspace.mit.edu/bitstream/handle/1721.1/8979/47210047-MIT.pdf? sequence=2 &isAllowed=y

[3] Krafes S, Chalh Z, Saka A (2018). A review on the control of second order underactuated mechanical systems. Complexity.

[4] Huang X, Ralescu AL, Gao H, Huang H (2019). A survey on the application of fuzzy systems for underactuated systems. Proc. Inst. Mech. Eng. Part I J. Syst. Control Eng..

[5] Yang T, Sun N, Fang Y (2022). Adaptive fuzzy control for a class of mimo underactuated systems with plant uncertainties and actuator deadzones: Design and experiments. IEEE Trans. Cybern..

[6] Nafa F, Boudouda A, Smaani B (2021). Adaptive wavelets sliding mode control for a class of second order underactuated mechanical systems. Acta Polytech..

[7] Zabihifar SH, Navvabi H, Yushchenko AS (2021). Dual adaptive neural network controller for underactuated systems. Robotica.

[8] Gupta N, Dewan L (2024). Adaptive neural network-based sliding mode control of rotary inverted pendulum system. J. Control Decis..

[9] Li N, Liu X, Liu C, He W, Wang H (2024). Adaptive stabilization control for a class of non-strict feedback underactuated nonlinear systems by backstepping. IEEE Trans. Autom. Sci. Eng..

[10] Gutierrez-Oribio, D., Stefanou, I. & Plestan, F. Passivity-based control of underactuated mechanical systems with coulomb friction: Application to earthquake prevention. arXiv preprint arXiv:2207.07181 (2022). 10.48550/arXiv.2207.07181.

[11] Franco E, Arpenti P, Donaire A, Ruggiero F (2024). Integral ida-pbc for underactuated mechanical systems subject to matched and unmatched disturbances. IEEE Control Syst. Lett..

[12] Gutiérrez-Oribio D, Mercado-Uribe JA, Moreno JA, Fridman L (2021). Robust global stabilization of a class of underactuated mechanical systems of two degrees of freedom. Int. J. Robust Nonlinear Control.

[13] Shah I (2023). Robust approach for global stabilization of a class of underactuated mechanical systems in presence of uncertainties. Complexity.

[14] Ovalle L, Ríos H, Llama M, Fridman L (2022). Continuous sliding-mode output-feedback control for stabilization of a class of underactuated systems. IEEE Trans. Autom. Control.

[15] Rehman FU, Mehmood N, Din SU, Mufti MR, Afzal H (2021). Adaptive sliding mode based stabilization control for the class of underactuated mechanical systems. IEEE Access.

[16] Nguyen T-V-A, Dong B-T, Bui N-T (2023). Enhancing stability control of inverted pendulum using Takagi-Sugeno fuzzy model with disturbance rejection and input-output constraints. Sci. Rep..

[17] Qaiser N, Iqbal N, Hussain A, Qaiser N (2007). Exponential stabilization of a class of underactuated mechanical systems using dynamic surface control. Int. J. Control Autom. Syst..

[18] Adıgüzel F, Yalçın Y (2022). Backstepping control for a class of underactuated nonlinear mechanical systems with a novel coordinate transformation in the discrete-time setting. Proc. Inst. Mech. Eng. Part I J. Syst. Control Eng..

[19] Rudra, S., Barai, R. K. & Maitra, M. Block backstepping design of nonlinear state feedback control law for underactuated mechanical systems (Springer, 2017). 10.1007/978-981-10-1956-2_3.

[20] Swaroop D, Hedrick JK, Yip PP, Gerdes JC (2000). Dynamic surface control for a class of nonlinear systems. IEEE Trans. Autom. Control.

[21] Yang Z, Dong C, Zhang X, Wang G (2022). Full-state time-varying asymmetric constraint control for non-strict feedback nonlinear systems based on dynamic surface method. Sci. Rep..

[22] Deng X, Yuan Y, Wei L, Xu B, Tao L (2022). Adaptive neural tracking control for nonstrict-feedback nonlinear systems with unknown control gains via dynamic surface control method. Mathematics.

[23] Ding F (2022). Dynamic surface control with a nonlinear disturbance observer for multi-degree of freedom underactuated mechanical systems. Int. J. Robust Nonlinear Control.

[24] Chen L, Wang Q, Hu C (2022). Adaptive fuzzy command filtered backstepping control for uncertain pure-feedback systems. ISA Trans..

[25] Chen M, Li Y, Wang H, Peng K, Wu L (2023). Adaptive fixed-time tracking control for nonlinear systems based on finite-time command-filtered backstepping. IEEE Trans. Fuzzy Syst..

[26] Hu J, Zhang D, Wu Z-G, Li H (2023). Neural network-based adaptive second-order sliding mode control for uncertain manipulator systems with input saturation. ISA Trans..

[27] Liu Z (2024). A novel faster fixed-time adaptive control for robotic systems with input saturation. IEEE Trans. Ind. Electron..

[28] Wu X, Zhao Y, Xu K (2021). Nonlinear disturbance observer based sliding mode control for a benchmark system with uncertain disturbances. ISA Trans..

[29] Adıgüzel F, Yalçın Y (2021). Discrete-time backstepping control with nonlinear adaptive disturbance attenuation for the inverted-pendulum system. Trans. Inst. Meas. Control.

[30] Wen T, Fang Y, Lu B (2022). Neural network-based adaptive sliding mode control for underactuated dual overhead cranes suffering from matched and unmatched disturbances. Auton. Intell. Syst..

[31] Hfaiedh A, Chemori A, Abdelkrim A (2022). Observer-based robust integral of the sign of the error control of class i of underactuated mechanical systems: Theory and real-time experiments. Trans. Inst. Meas. Control.

[32] Guo W, Liu D (2023). Adaptive neural network command filtered backstepping control for the underactuated tora system. IEEE Access.

[33] Wu X, Xu K, Ma M, Ke L (2021). Output feedback control for an underactuated benchmark system with bounded torques. Asian J. Control.

[34] Zhang Y (2023). Robust command-filtered control with prescribed performance for flexible-joint robots. IEEE Trans. Instrum. Meas..

[35] Liu Y, Zhang H, Wang Y, Liang H (2022). Adaptive containment control for fractional-order nonlinear multi-agent systems with time-varying parameters. IEEE/CAA J. Autom. Sin..

[36] Naderolasli A, Shojaei K, Chatraei A (2023). Finite-time velocity-free adaptive neural constrained cooperative control of Euler-Lagrange systems. Trans. Inst. Meas. Control.

[37] Naderolasli A, Shojaei K, Chatraei A (2023). Terminal sliding-mode disturbance observer-based finite-time adaptive-neural formation control of autonomous surface vessels under output constraints. Robotica.

[38] Naderolasli A, Shojaei K, Chatraei A (2023). Platoon formation control of autonomous underwater vehicles under Los range and orientation angles constraints. Ocean Eng..

[39] Guan A (2024). Experimental and modeling investigation on dynamic response of sticky control valves. Control Eng. Pract..

[40] Naderolasli A, Shojaei K, Chatraei A (2023). Leader-follower formation control of Euler-Lagrange systems with limited field-of-view and saturating actuators: A case study for tractor-trailer wheeled mobile robots. Eur. J. Control.

[41] Adıgüzel F, Yalçın Y (2022). Immersion and invariance disturbance observer-based nonlinear discrete-time control for fully actuated mechanical systems. Int. J. Syst. Sci..

